# Urinary heavy metal and trace element mixtures in chronic kidney disease: associations and implications for potential reverse causality

**DOI:** 10.3389/ftox.2026.1892052

**Published:** 2026-07-29

**Authors:** Sisi Xie, Julien Vaucher, Maïwenn Perrais, Aurélien Thomas, Pedro Marques-Vidal

**Affiliations:** 1 Department of Medicine, Internal Medicine, Lausanne University Hospital (CHUV) and University of Lausanne, Lausanne, Switzerland; 2 Department of Medicine and Specialties, Internal Medicine, Fribourg Hospital and University of Fribourg, Fribourg, Switzerland; 3 Faculty Unit of Toxicology, University Center of Legal Medicine Lausanne-Geneva, Lausanne University Hospital and University of Lausanne, Lausanne, Switzerland; 4 Unit of Forensic Toxicology and Chemistry, CURML, Lausanne and Geneva University Hospitals, Lausanne, Switzerland

**Keywords:** chronic kidney disease, heavy metals, reverse causality, trace elements, urinary biomarkers

## Abstract

**Introduction:**

Chronic kidney disease (CKD) is a growing public health challenge, yet impaired kidney function may substantially alter urinary biomarker profiles, complicating their interpretation. We aimed to investigate the associations between urinary element concentrations and prevalent CKD, and to explore whether impaired kidney function and altered urinary excretion may contribute to reverse causality in the interpretation of urinary biomarkers.

**Methods:**

We conducted a cross-sectional analysis of urinary element mixtures and prevalent CKD among 6,192 adults (51.7% female; mean age 52.4 ± 10.7 years). Associations between urinary element concentrations and prevalent CKD were evaluated using logistic regression, restricted cubic spline models, and three mixture modeling approaches, including Bayesian kernel machine regression, weighted quantile sum regression, and quantile g-computation.

**Results:**

Participants with CKD exhibited lower urinary concentrations of selenium, molybdenum, cadmium, mercury, lead, and thallium, whereas urinary zinc concentrations were higher. Non-linear associations were observed for several elements. Across multiple mixture models, higher combined urinary element concentrations were consistently associated with lower odds of prevalent CKD. Cadmium, mercury, selenium, and thallium contributed most strongly to these inverse associations, whereas zinc showed a positive contribution.

**Discussion:**

These findings are unlikely to reflect protective effects and may instead reflect impaired urinary excretion resulting from kidney dysfunction. Our findings suggest that urinary elements in CKD reflect not only exposure but also disease-related alterations in renal excretory function. These findings highlight the potential for reverse causality when interpreting urinary elements as biomarkers or predictors of kidney outcomes.

## 1 Introduction

Chronic kidney disease (CKD) is a major global public health problem, characterized by a progressive decrease in estimated glomerular filtration rate (eGFR) and proteinuria due to permanent loss of nephrons (Kalantar-Zadeh et al., 2021). According to the Global Burden of Disease Study, approximately 697.5 million people were affected by CKD globally in 2017 (Collaboration, 2020). While traditional risk factors such as diabetes, hypertension, and glomerulonephritis are becoming increasingly prevalent, accumulating evidence also suggests that chronic exposure to environmental pollutants such as heavy metals may impair kidney function (Kim et al., 2015).

The kidney is the main target organ of heavy metals, which tend to accumulate following environmental exposure. Existing studies have shown that lead, cadmium, mercury, and arsenic are common toxic heavy metals that can significantly damage kidney function (Ekong et al., 2006; Tsai et al., 2017). Conversely, essential trace elements such as selenium, manganese, and zinc are involved in kidney physiology and redox homeostasis, although their roles in CKD appear complex and context dependent (Xie et al., 2022). Emerging evidence further suggests that molybdenum homeostasis may be linked to systemic disease processes beyond the kidney, as higher circulating molybdenum concentrations have been associated with increased risks of certain cancers, highlighting the multi-organ implications of disturbed trace element balance (Matuszczak et al., 2024). Previous studies, many of which are cross-sectional, have explored the associations between individual elements and CKD. However, few have accounted for the fact that elements are typically encountered as mixtures, nor have they modeled potential non-linear relationships or accounted for the possibility that impaired kidney function may itself alter urinary element concentration, complicating assessment. These factors are particularly relevant when interpreting urinary element concentrations, which reflect both external exposure and internal physiological factors, including kidney filtration and retention.

In a previous prospective analysis conducted in the same population and including the same panel of urinary elements, we found that higher baseline urinary element concentrations of vanadium, cobalt, molybdenum, and other elements were associated with incident CKD over a 12.5-year follow-up period (Xie et al., 2025). That analysis focused on longitudinal associations and long-term risk. In contrast, the current cross-sectional study was intentionally designed to address a different but complementary set of questions: How is current kidney function associated with urinary element patterns? Do non-linear or threshold effects exist? Are there synergistic or antagonistic interactions within element mixtures? And importantly, does impaired kidney filtration or retention in CKD alter the interpretation of urinary biomarker data?

To address these questions, we analyzed urinary concentrations of 23 heavy metals and trace elements in a general population cohort, using three mixture modeling approaches, Bayesian kernel machine regression (BKMR), weighted quantile sum (WQS) regression, and quantile g-computation (qgcomp), alongside restricted cubic spline (RCS) analysis. Our findings aim to provide insight into the complex interplay between urinary heavy metals and trace elements and kidney function, and to complement existing prospective evidence by exploring urinary concentration patterns and the interpretive challenges they pose in the context of CKD. For simplicity, the term “elements” is used throughout this manuscript to collectively refer to heavy metals and trace elements.

## 2 Materials and methods

### 2.1 Data source and study population

The CoLaus|PsyCoLaus study is a population-based cohort initiated in 2003 to investigate the epidemiology and genetic determinants of cardiovascular risk factors in Lausanne, Switzerland. Participants were randomly selected from the population registry of Lausanne. Eligible participants were adults aged 35–75 years of Caucasian origin who provided written informed consent (Firmann et al., 2008). The present study used data from 6,733 baseline participants recruited between 2003 and 2006. Exclusion criteria specific to the present analysis are described in Section 2.5.

### 2.2 Measurement of 23 elements in urine

Spot urine samples were collected in the morning following an overnight fast. Samples were aliquoted and immediately stored at −80 °C until analysis. All urinary element concentrations were corrected for urine dilution using urinary creatinine (μg/g creatinine). Specific gravity was not available in this study and could not be used for dilution adjustment. The measurement of 23 elements (silver (Ag), aluminium (Al), arsenic (As), beryllium (Be), bismuth (Bi), cadmium (Cd), cobalt (Co), chromium (Cr), copper (Cu), iron (Fe), mercury (Hg), iodine (I), lithium (Li), manganese (Mn), molybdenum (Mo), nickel (Ni), lead (Pb), antimony (Sb), selenium (Se), tin (Sn), thallium (Tl), vanadium (V) and zinc (Zn)) in urine was performed as described in our previous study (Xie et al., 2025). Briefly, urine samples (200 µL) were diluted with 1.8 mL of 1% nitric acid (HNO_3_) solution containing rhodium (10 ng/mL) and indium (10 ng/mL) as internal standards. Samples were analyzed using an inductively coupled plasma mass spectrometer (ICP-MS; 7800 Series, Agilent Technologies). Quantitative analysis included 23 elements, and details of calibration, quality control, and limit of detection (LOD) assessments were as described previously (Xie et al., 2025). As spot urine samples were used, urinary concentrations may represent different exposure windows depending on the element. Elements with long biological half-lives, particularly cadmium, may reflect longer-term body burden and kidney accumulation, whereas essential trace elements with tighter homeostatic regulation and faster turnover may primarily reflect more recent intake and short-term excretory dynamics. Before statistical analyses, element concentrations below the limit of detection (LOD) were replaced with one-half of the corresponding LOD (LOD/2). Elements with more than 75% of measurements below the LOD were excluded *a priori* because the limited number of detectable values could lead to unstable effect estimation in subsequent regression and mixture modeling analyses.

### 2.3 Outcome assessment

The primary outcome of this study was study-defined CKD. Serum creatinine levels were measured using the Jaffe method to estimate the glomerular filtration rate. eGFR was calculated according to the 2021 Chronic Kidney Disease Epidemiology Collaboration (CKD-EPI) equation (Inker et al., 2021). In the present study, CKD was operationally defined as an eGFR <60 mL/min/1.73 m^2^ at the baseline visit (Levey et al., 2011). Because eGFR was measured only at scheduled survey visits, persistence of reduced eGFR for at least 3 months could not be confirmed according to the Kidney Disease: Improving Global Outcomes (KDIGO) criteria. Therefore, CKD in this study represents an epidemiological definition based on a single baseline assessment and should not be interpreted as a clinical diagnosis. Throughout the manuscript, the term “CKD” refers to this study-defined outcome unless otherwise specified.

### 2.4 Covariates

The covariates were selected *a priori* based on previous literature and biological plausibility, including established demographic and clinical risk factors for chronic kidney disease.

The covariates included in the analysis were sex (male/female), age (years), marital status (living alone/living in a couple), education (categorized as low, medium, or high), smoking status (Never/Former/Current), weekly alcohol consumption (reported in units), hypertension (yes/no), diabetes (yes/no), and body mass index (BMI) (normal, overweight, or obese). Detailed descriptions of the covariates, including their definitions, measurement methods, and classification criteria, are provided in the Supplementary Material.

### 2.5 Exclusion criteria

Participants were excluded from the analysis if they had missing data for any of the following: (1) elements, (2) eGFR, or (3) the covariates mentioned above, or if they were receiving iodine or lithium treatment.

### 2.6 Ethics approval and consent to participate

This study is part of the CoLaus|PsyCoLaus cohort, which was ethically approved by the institutional Ethics Committee of the University of Lausanne (subsequently renamed as the Ethics Commission of Canton Vaud, www.cer-vd.ch) under project number PB_2018-00038 and reference 239/09. All participants provided written informed consent after receiving a detailed explanation of the study’s aims and procedures, in accordance with the principles of the Declaration of Helsinki.

### 2.7 Statistical analysis

Statistical analyses were conducted using Stata v.18 (Stata Corp, College Station, TX, United States) and RStudio Desktop (Version 2024.04.2 + 764). Categorical variables were summarized as counts and percentages, while continuous variables were presented as mean ± standard deviation. Group comparisons were evaluated using the chi-square test for categorical variables and the Student’s t-test for continuous variables.

All urinary element concentrations were analyzed as creatinine-adjusted values (expressed as micrograms per gram of creatinine, μg/g creatinine), as described above. To approximate normality, all concentrations were log-transformed before analysis. Accordingly, odds ratios (ORs) estimated for continuous exposure variables represent a one-unit increase in the natural logarithm of urinary element concentration, corresponding to an approximately 2.72-fold increase in the original concentration. Subsequently, we applied the least absolute shrinkage and selection operator (LASSO) regression for variable selection. Given the limited number of prevalent CKD cases relative to the number of urinary elements and the multi-element exposure setting, LASSO was used as an exploratory screening tool to reduce model complexity and improve model stability rather than for formal post-selection inference. The resulting parsimonious set of elements was carried forward to subsequent single-element, non-linear, and mixture analyses. Because LASSO was used for exploratory variable screening, downstream regression and mixture analyses should be interpreted as exploratory rather than confirmatory. Pairwise Pearson correlation coefficients were calculated to characterize the correlation structure among the selected urinary elements before subsequent regression and mixture analyses. The optimal regularization parameter (λ) was determined using k-fold cross-validation, and variables with non-zero coefficients at the cross-validated optimal λ were retained for downstream analyses.

Based on the selected elements, we first conducted multivariable-adjusted logistic regression analyses using both continuous variables and quartile groupings. A trend test (p-trend) was used to assess linear dose–response relationships across quartiles. Multicollinearity among covariates included in the multivariable regression models was assessed using variance inflation factors (VIFs), and no evidence of problematic multicollinearity was observed (mean VIF = 1.35; maximum VIF = 1.8). To further explore the non-linear associations between urinary concentrations and CKD status, we applied RCS regression with four knots at the fifth, 35th, 65th, and 95th percentiles of the exposure distribution, following commonly recommended practice. Model fit and evidence of non-linearity were evaluated using likelihood ratio tests comparing models with and without spline terms.

To investigate the joint effects of multiple urinary element concentrations on kidney function, we employed three complementary mixture modeling approaches: WQS regression, qgcomp, and BKMR. For WQS regression, separate positive and negative directional models were fitted by specifying the assumed direction of the mixture effect during weight estimation (b1_pos = TRUE or FALSE). Exposures were categorized into quartiles (q = 4), and models were fitted using a 40% training dataset and a 60% validation dataset with 1,000 bootstrap samples and a fixed random seed of 100. No repeated holdout procedure was used. Elements with mean weights ≥0.10 were considered important contributors only for statistically significant directional models, representing a commonly used heuristic threshold that exceeds the uniform weight (1/p ≈ 0.056, given p = 18 mixture components) expected under equal contribution. For qgcomp, we applied quantile g-computation assuming additive mixture effects, using quartiles (q = 4) and a generalized linear model with a binomial family and logit link. Overall mixture effects were estimated using 500 bootstrap replications, with a fixed random seed of 123 to ensure reproducibility. For BKMR, exposures were modeled jointly to allow for non-linear and interactive effects among elements. Detailed BKMR model specifications, including Markov chain Monte Carlo (MCMC) settings, variable-selection settings, prior specifications, convergence assessment, and effective sample size estimates, are provided in the Supplementary Material.

Additionally, to assess the robustness of our findings and account for potential biases introduced by creatinine adjustment, especially in individuals with impaired kidney function, we first conducted a sensitivity analysis using non-creatinine-adjusted urinary element concentrations. We then performed an additional sensitivity analysis in which non-creatinine-adjusted urinary element concentrations were analysed with urinary creatinine included as a continuous covariate to account for urine dilution. We further conducted a sensitivity analysis comparing participants with CKD to a strictly defined healthy control group (eGFR≥90 mL/min/1.73 m^2^ and without diabetes or hypertension) to evaluate the robustness of the observed associations. Complementary multivariable linear regression analyses were performed with log-transformed urinary element concentrations as the dependent variables and CKD status as the independent variable, adjusting for the same covariates as the primary analyses. To assess the potential influence of selection due to complete-case exclusion, we additionally performed an inverse probability weighted sensitivity analysis. Stabilized inverse probability weights were estimated based on the probability of inclusion in the final analytic sample using observed baseline characteristics, and the weighted single-element logistic regression models were adjusted for the same covariates as the primary analyses.

A two-sided *p* value < 0.05 was considered statistically significant. Given the exploratory nature of the analyses and the large number of statistical tests performed, individual P values were interpreted cautiously, and the findings should be regarded as hypothesis-generating rather than confirmatory.

## 3 Results

### 3.1 Study design and population

We excluded participants who had missing data on 23 elements (n = 329), eGFR (n = 11), any of the covariates (n = 19), and those receiving iodine or lithium treatments (n = 182). Finally, 6,192 participants were included in this study (Supplementary Figure S1). Among the included participants, 171 (2.8%) met the study definition of CKD. The baseline distribution of participants across the Kidney Disease: Improving Global Outcomes (KDIGO) glomerular filtration rate (GFR) categories is shown in Supplementary Table S1. 51.7% of the total population was female. As shown in Table 1, compared with participants in the non-CKD group, the CKD group was older, had a higher prevalence of obesity, hypertension, and diabetes, higher serum creatinine and uric acid values, but had fewer current smokers. Median urinary creatinine concentrations were lower in participants with CKD than in those without CKD. To evaluate the potential impact of participant exclusion on the representativeness of the analytical sample, baseline characteristics were compared between included and excluded participants (Supplementary Table S2). Significant differences were observed in age, sex, alcohol status, hypertension, and eGFR between the two groups.

**TABLE 1:** Characteristics of participants by categories of kidney function. CoLaus|PsyCoLaus study, Lausanne, Switzerland.

Variables	Total (N = 6,192)	Non-CKD (N = 6,021)	CKD (N = 171)	*P-value*
Age, years	52.4 ± 10.7	52.1 ± 10.5	64.2 ± 9.4	**<0.001**
Female, %	3202 (51.7)	3106 (51.6)	96 (56.1)	0.240
Education level, %	​	​	​	0.233
High	1225 (19.8)	1199 (19.9)	26 (15.2)	​
Middle	1505 (24.3)	1457 (24.2)	48 (28.1)	​
Low	3462 (55.9)	3365 (55.9)	97 (56.7)	​
Marital status, %	​	​	​	0.069
Living alone	2028 (32.8)	1961 (32.6)	67 (39.2)	​
Living in couple	4164 (67.2)	4060 (67.4)	104 (60.8)	​
Smoking status, %	​	​	​	**0.001**
Never	2494 (40.3)	2422 (40.2)	72 (42.1)	​
Former	2014 (32.5)	1942 (32.3)	72 (42.1)	​
Current	1684 (27.2)	1657 (27.5)	27 (15.8)	​
Alcohol consumption, %	​	​	​	0.167
None	1734 (28.0)	1675 (27.8)	59 (34.5)	​
1–13/week	3362 (54.3)	3272 (54.4)	90 (52.6)	​
14–27/week	856 (13.8)	839 (13.9)	17 (10.0)	​
28+/week	240 (3.9)	235 (3.9)	5 (2.9)	​
BMI groups, %	​	​	​	**<0.001**
Normal	2989 (48.3)	2938 (48.8)	51 (29.8)	​
Overweight	2262 (36.5)	2183 (36.3)	79 (46.2)	​
Obese	941 (15.2)	900 (14.9)	41 (24.0)	​
Hypertension, %	2257 (36.5)	2137 (35.5)	120 (70.2)	**<0.001**
Diabetes, %	402 (6.5)	374 (6.2)	28 (16.4)	**<0.001**
Serum creatinine, µmol/L	79.8 ± 19.7	78.4 ± 13.5	126.4 ± 73.9	**<0.001**
Urinary creatinine, mg/dL	143 (100–192)	144 (101–193)	122 (84–163)	**<0.001**
Uric acid, µmol/L	312.4 ± 84.8	310.1 ± 83.1	394.3 ± 102.5	**<0.001**
eGFR, ml/min/1.73 m^2^	89.8 ± 15.1	90.9 ± 13.8	51.7 ± 9.2	**<0.001**

For categorical variables, results were reported as the number of participants along with column percentages. For continuous variables, data were summarized as mean values with standard deviations. Statistical analyses included chi-square tests for the comparison of categorical variables and Student’s t-tests for continuous variables. Urinary creatinine is presented as median (interquartile range); *P-*values were obtained using the Wilcoxon rank-sum test. Abbreviations: BMI, body mass index; CKD, chronic kidney disease; eGFR, estimated glomerular filtration rate. Bold values indicate statistical significance (*P* < 0.05).

### 3.2 Element preprocessing

In this study, 23 elements were initially included for analysis. Four elements with more than 75% of values below the LOD (Be: 77.75%, Mn: 88%, Ag: 82.19%, Bi: 97.50%) were excluded during data preprocessing. Supplementary Table S3 details the number and percentage of participants with each element below the LOD. Among the remaining 19 elements, one (Ni) was excluded based on LASSO regression, leaving 18 elements for all subsequent analyses. Supplementary Table S4 shows the distribution of creatinine-adjusted urinary concentrations for all 23 elements, including the median and interquartile range. The distributions of the final selected elements included in the main analyses are summarized in Supplementary Table S5.

### 3.3 Correlation analysis of urinary elements

Pairwise Pearson correlation coefficients among urinary elements showed generally weak correlations. The strongest observed correlations were between Cd and Cu (r = 0.35), Cr and V (r = 0.34), Mo and Se (r = 0.34), and Pb and Cr (r = 0.34). Most other element pairs showed correlations below r = 0.30, indicating generally weak pairwise correlations among urinary elements (Supplementary Figure S2). These findings characterize the overall correlation structure among urinary elements and provide descriptive context for the subsequent mixture analyses.

### 3.4 Associations between individual urinary elements and CKD: logistic regression

Table 2 shows the association between individual urinary element concentrations and CKD based on multivariable logistic regression. In continuous models, higher urinary concentrations of Se, Mo, Cd, Hg, Pb, and Tl were associated with lower odds of CKD. To assess dose-response relationships, concentrations were categorized into quartiles, with Q1 as the reference. Compared to the lowest quartile, participants in the highest quartile of Zn had higher odds of having CKD (odds ratio (OR) = 1.92, 95% CI: 1.14, 3.23). In contrast, participants in the highest quartiles of Se, Mo, Cd, Hg, Pb, and Tl had lower odds of having CKD, with ORs of 0.40 (95% CI: 0.25, 0.63), 0.61 (0.38, 0.99), 0.38 (0.21, 0.68), 0.32 (0.19, 0.54), 0.26 (0.16, 0.42), and 0.39 (0.25, 0.60), respectively. Trend tests were consistent with the quartile-based findings, supporting dose–response patterns across quartiles.

**TABLE 2:** Associations of single elements with CKD status, CoLaus|PsyCoLaus study, Lausanne, Switzerland.

Elements	Continuous OR (95% CI)	Q1	Q2 OR (95% CI)	Q3 OR (95% CI)	Q4 OR (95% CI)	*P for trend*
Li	0.96 (0.80, 1.16)	1.00 (ref.)	1.00 (0.63, 1.58)	0.87 (0.55, 1.38)	0.97 (0.62, 1.52)	0.989
Al	0.99 (0.84, 1.18)	1.00 (ref.)	1.36 (0.85, 2.19)	1.08 (0.67, 1.74)	1.23 (0.77, 1.95)	0.703
V	0.77 (0.58, 1.02)	1.00 (ref.)	1.00 (0.63, 1.58)	0.87 (0.55, 1.38)	0.97 (0.62, 1.52)	0.052
Cr	1.05 (0.88, 1.26)	1.00 (ref.)	0.94 (0.59, 1.48)	0.69 (0.42, 1.11)	1.00 (0.65, 1.54)	0.756
Co	1.04 (0.85, 1.26)	1.00 (ref.)	0.87 (0.54, 1.40)	0.64 (0.39, 1.06)	1.00 (0.61, 1.63)	0.806
Cu	0.94 (0.65, 1.35)	1.00 (ref.)	0.79 (0.48, 1.29)	0.57 (0.34, 0.94)	0.72 (0.45, 1.16)	0.229
Zn	1.28 (0.98, 1.65)	1.00 (ref.)	1.62 (0.93, 2.81)	1.73 (1.01, 2.96)	1.92 (1.14, 3.23)	**0.018**
As	0.99 (0.86, 1.14)	1.00 (ref.)	1.16 (0.76, 1.78)	0.97 (0.62, 1.53)	1.07 (0.69, 1.66)	0.971
Se	**0.28 (0.16, 0.48)**	1.00 (ref.)	0.73 (0.49, 1.09)	0.4 (0.26, 0.64)	0.40 (0.25, 0.63)	**<0.001**
Mo	**0.65 (0.50, 0.86)**	1.00 (ref.)	0.98 (0.65, 1.48)	0.75 (0.48, 1.17)	0.61 (0.38, 0.99)	**0.014**
Cd	**0.56 (0.42, 0.75)**	1.00 (ref.)	1.24 (0.76, 2.04)	0.77 (0.46, 1.31)	0.38 (0.21, 0.68)	**<0.001**
Sn	1.09 (0.90, 1.31)	1.00 (ref.)	1.04 (0.65, 1.67)	0.93 (0.58, 1.51)	1.02 (0.65, 1.62)	0.971
Sb	0.87 (0.69, 1.09)	1.00 (ref.)	0.98 (0.63, 1.52)	1.05 (0.68, 1.63)	0.86 (0.54, 1.37)	0.641
Hg	**0.70 (0.57, 0.85)**	1.00 (ref.)	0.83 (0.56, 1.22)	0.55 (0.35, 0.85)	0.32 (0.19, 0.54)	**<0.001**
Pb	**0.49 (0.37, 0.65)**	1.00 (ref.)	0.62 (0.41, 0.94)	0.24 (0.15, 0.40)	0.26 (0.16, 0.42)	**<0.001**
Tl	**0.44 (0.31, 0.61)**	1.00 (ref.)	0.52 (0.34, 0.79)	0.41 (0.26, 0.64)	0.39 (0.25, 0.60)	**<0.001**
Fe	1.00 (0.83, 1.22)	1.00 (ref.)	1.09 (0.68, 1.73)	0.84 (0.52, 1.34)	0.99 (0.62, 1.56)	0.766
I	1.16 (0.90, 1.49)	1.00 (ref.)	1.29 (0.80, 2.08)	0.96 (0.58, 1.58)	1.05 (0.65, 1.70)	0.821

Multivariable logistic regression models were used for the analysis. Results are presented as odds ratios (ORs) with corresponding 95% confidence intervals and P values for trends. For continuous exposure variables, ORs, represent a one-unit increase in the natural logarithm of urinary element concentration, corresponding to an approximately 2.72-fold increase in the original concentration. The models were adjusted for the following variables: age (continuous), sex (male or female), BMI (normal, overweight, or obese), education (low, medium, high), marital status (alone or in a couple), smoking status (never, former, current), alcohol consumption (categorized into four groups), hypertension (yes or no), and diabetes (yes or no). Abbreviations: Continuous: log-transformed, creatinine-adjusted urinary concentrations of elements; OR, odds ratio; Q, quartiles; CKD, chronic kidney disease.

Bold values indicate statistical significance (*P* < 0.05).

**FIGURE 1:**
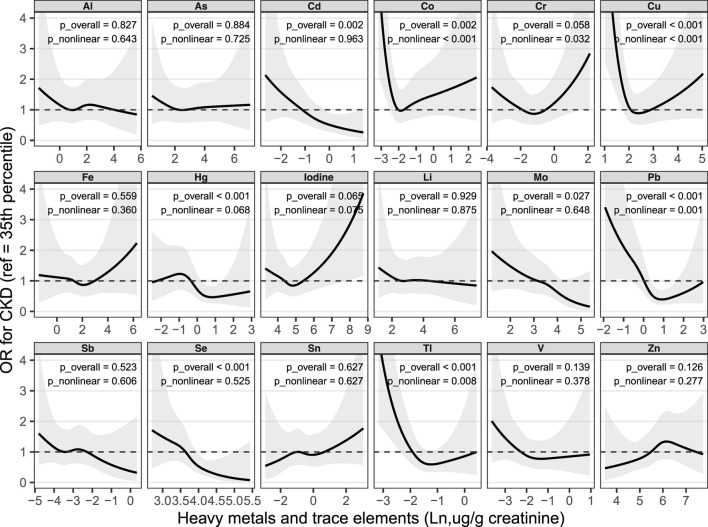
Restricted cubic spline models of urinary elements and CKD. CoLaus study, Lausanne, Switzerland. Dose–response relationships between log-transformed, creatinine-adjusted urinary concentrations of selected elements and CKD status. Models were adjusted for the following variables: age (continuous), sex (male or female), BMI (normal, overweight, or obese), education (low, medium, high), marital status (alone or in a couple), smoking status (never, former, current), alcohol consumption (categorized into four groups), hypertension (yes or no), and diabetes (yes or no). Solid lines represent estimated ORs; shaded areas indicate 95% confidence intervals. The reference point was set at the 35th percentile of the element concentration distribution.

To further investigate potential non-linear dose–response relationships, RCS models were applied to each element. As shown in Figure 1, Cd, Co, Cu, Hg, Mo, Pb, Se, and Tl showed significant overall associations with CKD status. In addition, Co, Cr, Cu, Pb, and Tl exhibited statistically significant non-linear concentration–response relationships (*p* for non-linear<0.05). Specifically, the significant non-linear associations for Co, Cr, Cu, Pb, and Tl generally followed U-shaped or threshold-like patterns, characterized by a steep decline at lower concentrations, a nadir at intermediate levels, and a gradual increase at higher concentrations. These findings provide complementary evidence to the logistic regression results and highlight complex, element-specific concentration–response patterns across levels of kidney function.

### 3.5 Associations between urinary element mixture profiles and CKD status: WQS, qgcomp, and BKMR models

#### 3.5.1 WQS regression analysis

**FIGURE 2:**
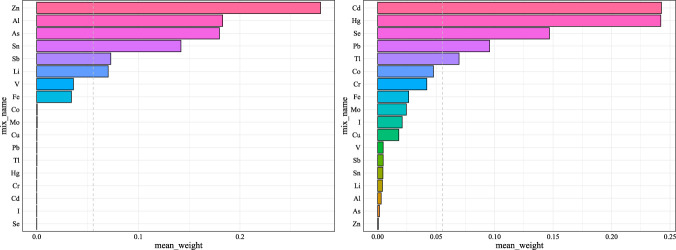
Association of urinary element mixtures with CKD status by WQS analyses. CoLaus study, Lausanne, Switzerland. WQS regression identifying key urinary elements associated with CKD status. Left panel: elements contributing positively; right panel: elements contributing negatively. Bars represent mean weights across bootstrap samples. Elements with weights ≥0.10 were considered major contributors only for statistically significant directional models. Weights from non-significant directional models are shown for descriptive purposes only.

We separately modeled negative and positive associations using directional WQS regression. The model for negative associations revealed a significant inverse association between the WQS index and CKD status (estimate = −0.94, 95% CI [−1.35, −0.53], *p* < 0.001), indicating an inverse association between the overall mixture index and CKD status. In this model, the most influential contributors (weights≥0.10) were Cd (0.24), Hg (0.24), Se (0.15), and Pb (0.10), as shown in Figure 2. The positive directional WQS model was not statistically significant (estimate = −0.15, 95% CI: −0.54 to 0.25, *p* = 0.474). Therefore, although Zn, Al, As, and Sn received relatively higher weights in the positive directional model, these weights are presented for descriptive purposes only and were not interpreted as evidence of important contributors.

#### 3.5.2 qgcomp *analysis*

**FIGURE 3:**
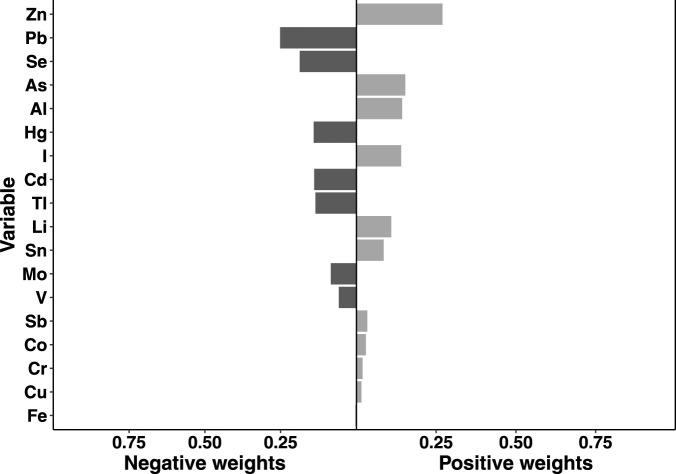
Association of urinary element mixtures with CKD status by qgcomp analyses. CoLaus study, Lausanne, Switzerland. Qgcomp weights identifying key urinary elements associated with CKD status. Bars indicate each element’s contribution to the overall mixture effect, with leftward (negative) and rightward (positive) weights representing the direction of association, not causal direction. Elements with weights ≥0.10 were considered major contributors.

Results from qgcomp also indicated lower overall mixture index values among participants with CKD, reflected by a significant negative association between urinary element mixtures and CKD status (estimate = −0.64, 95% CI: [−1.02, −0.26], *p* < 0.001). Based on the weight of individual elements, Pb (0.25) was identified as a key contributor to the negative mixture effect, while Zn (0.27) contributed positively to the overall mixture effect, as shown in Figure 3.

#### 3.5.3 BKMR analysis

**FIGURE 4:**
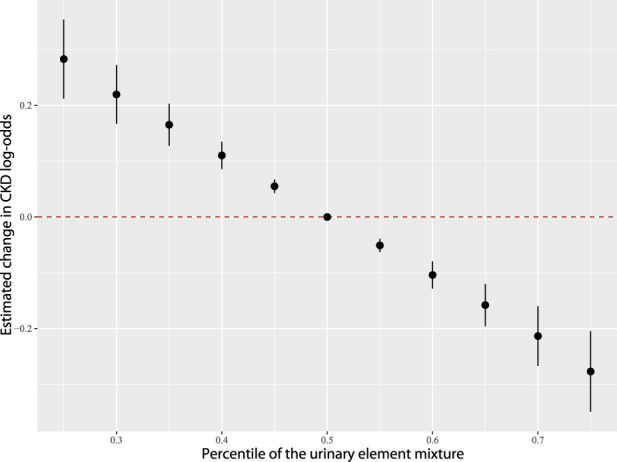
Overall association of urinary element mixtures with CKD status in the BKMR model. CoLaus study, Lausanne, Switzerland. Overall association between the urinary element mixture and CKD status estimated using the BKMR model. The x-axis represents percentiles of the urinary element mixture based on standardized log-transformed, creatinine-adjusted urinary element concentrations, with all elements jointly fixed at the same percentile and compared with the reference level at the 50th percentile. The y-axis shows the estimated change in CKD log-odds. Error bars denote 95% credible intervals.

**FIGURE 5:**
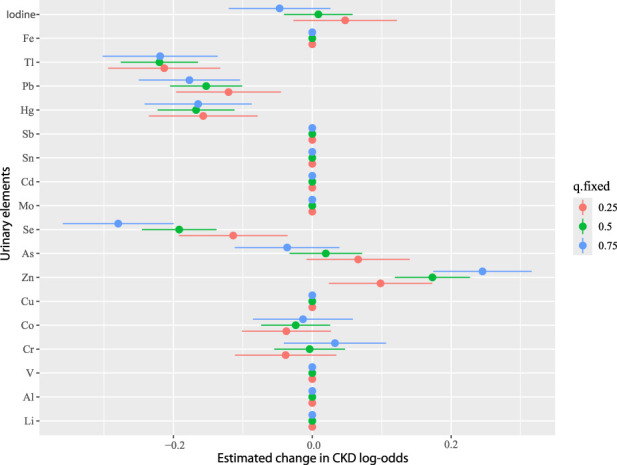
Estimated single-element effects of urinary element concentrations on CKD status from the BKMR model. CoLaus study, Lausanne, Switzerland. Single-element effect estimates for urinary elements on CKD status based on the BKMR model. Each point represents the estimated change in CKD log-odds associated with an increase in a given element from its 25th to 75th percentile, while holding all other elements fixed at the 25th (red), 50th (green), or 75th (blue) percentile. Horizontal lines denote 95% credible intervals.

Figure 4 shows a significant overall negative association between urinary element mixture profiles and CKD status. In the BKMR model, elements with a posterior inclusion probability ≥0.5 included Cr, Zn, Se, Hg, Pb, Tl, and I. Further analysis, where the concentrations of other elements were fixed at the 25th, 50th, and 75th percentiles, revealed lower urinary concentrations of Tl, Pb, Hg, and Se among participants with CKD, whereas Zn showed higher concentrations (Figure 5). The univariate concentration–response analysis indicated heterogeneous, non-flat patterns for a subset of elements (e.g., Se, Pb, Zn, Tl), whereas most others showed minimal marginal effects (Supplementary Figure S3). Meanwhile, the bivariate dose-response analysis did not identify significant interactions between elements (Supplementary Figure S4). Overall, these findings suggest that urinary element mixtures exhibit complex, non-uniform associations with CKD status, with individual elements potentially exhibiting opposing or similar effects across different urinary concentration ranges.

### 3.6 Sensitivity and additional analyses

Sensitivity analyses using non-creatinine-adjusted urinary element concentrations yielded results largely consistent with the primary analyses (Supplementary Table S6), with lower urinary concentrations of Cd, Pb, Hg, and Tl remaining significantly associated with CKD status. Similar results were observed when non-creatinine-adjusted urinary element concentrations were analyzed with urinary creatinine included as a continuous covariate (Supplementary Table S7). Lower urinary concentrations of Se, Mo, Cd, Hg, Pb, and Tl remained significantly associated with CKD status, with no material changes in the direction or magnitude of the associations compared with the primary creatinine-standardized analyses. These findings indicate that the primary associations were robust to an alternative urine dilution adjustment strategy and were not solely attributable to creatinine ratio standardization. Restricting the control group to participants with preserved kidney function (eGFR ≥90 mL/min/1.73 m^2^) and without hypertension or diabetes did not materially alter the observed associations (Supplementary Table S8). In complementary multivariable linear regression analyses, in which log-transformed urinary element concentrations were treated as the dependent variables and CKD status as the independent variable, CKD was significantly associated with lower urinary concentrations of Se, Mo, Cd, Hg, Pb, and Tl and higher urinary Zn concentrations (Supplementary Table S9). Results from the inverse-probability-weighting sensitivity analysis were materially consistent with those of the primary complete-case analyses (Supplementary Table S10). The direction and magnitude of the observed associations remained largely unchanged, suggesting that complete-case exclusion was unlikely to have materially influenced the primary findings.

## 4 Discussion

In this large cross-sectional study, we comprehensively evaluated the associations between urinary concentrations of elements and CKD status, employing single-element logistic regression, non-linear concentration–response analyses, and multiple mixture modeling approaches. We identified seven elements that were significantly associated with CKD status. Inverse associations with CKD were observed for urinary Se, Mo, Cd, Hg, Pb, and Tl in both continuous and categorical analyses. In contrast, Zn showed a positive association with CKD in the categorical analyses. These findings were further supported by RCS analyses, which revealed non-linear concentration–response patterns for several elements, and by mixture models (WQS, qgcomp, and BKMR), which consistently demonstrated lower overall urinary mixture profiles among participants with CKD. Within the mixture context, Pb emerged as a leading contributor to the negative association. Zn showed positive contributions in qgcomp and BKMR, whereas the positive directional WQS model was not statistically significant and was therefore not interpreted. Although WQS regression, qgcomp, and BKMR consistently supported an overall inverse association between the urinary element mixture and CKD, the relative importance of individual elements differed across methods. This is expected because the three approaches rely on different statistical assumptions and quantify mixture effects in different ways. Specifically, WQS estimates a weighted mixture index under a constrained directional assumption, qgcomp estimates additive overall mixture effects based on quantized exposures, whereas BKMR allows for potential nonlinear and interactive associations while evaluating variable importance using posterior inclusion probabilities. Therefore, consistency in the overall mixture association across methods was considered more informative than complete agreement in the ranking of individual elements. Importantly, the present study suggests that urinary element concentrations in individuals with established CKD should be interpreted as reflecting both environmental exposure and altered renal excretory function, rather than exposure alone. This finding highlights the importance of considering kidney function when interpreting urinary element biomarkers.

These findings offer complementary insights to our previous prospective analysis using the same cohort, which focused on baseline urinary elements as predictors of incident kidney function decline over time. In contrast, the current study explores how existing CKD status is associated with urinary concentrations of elements, thereby addressing a fundamentally different scientific question. Whereas the previous study primarily evaluated urinary elements as biomarkers of environmental exposure, the present study emphasizes how impaired kidney function influences urinary element concentrations and their interpretation. This difference in temporal framework may help explain why some previous studies reported positive associations between urinary heavy metal exposure and CKD risk, whereas inverse associations were observed in the present cross-sectional analysis. In prospective or exposure-oriented studies, higher urinary metal concentrations may reflect greater environmental exposure before the development of kidney dysfunction. However, among individuals with established CKD, reduced filtration and altered tubular handling may decrease urinary elimination of certain elements despite unchanged or increased systemic burden, leading to lower urinary concentrations and apparent inverse associations. The observed inverse associations for nephrotoxic metals such as Cd, Hg, and Pb may therefore reflect reduced kidney clearance or altered retention in individuals with impaired kidney function, rather than indicating any protective effect. Accordingly, the present findings should be regarded as complementary to, rather than an independent confirmation of, our previous prospective results. Together, the two studies provide a more complete picture of the bidirectional relationships between urinary element concentrations and kidney health across disease stages.

An important methodological consideration when interpreting these findings is the use of spot urine samples and creatinine-adjusted urinary concentrations. Depending on their toxicokinetic properties and biological half-lives, urinary biomarkers may represent different exposure windows. Elements with long biological half-lives may better reflect long-term body burden and kidney accumulation, whereas essential trace elements with tighter homeostatic regulation and faster turnover may be more strongly influenced by recent intake and short-term excretory dynamics.

In individuals with CKD, impaired kidney function may further complicate the interpretation of urinary biomarkers. Reduced glomerular filtration and tubular dysfunction can lead to decreased urinary excretion of certain elements despite unchanged or increased systemic burden. Although impaired kidney function may provide a biologically plausible explanation for the observed associations, differences in environmental exposure, dietary intake, medication use, muscle mass, tubular handling, and other physiological processes may also contribute to the observed urinary element profiles. Therefore, the inverse associations observed in this cross-sectional study should be interpreted cautiously and may reflect disease-related changes in urinary excretion rather than causal protective effects.

Consistent with this interpretation, we observed lower urinary creatinine concentrations among participants with CKD compared with those without CKD, indicating that creatinine standardization may not be fully comparable across levels of kidney function. While alternative approaches to account for urine dilution, such as specific gravity or covariate-adjusted methods, have been proposed, these approaches also rely on assumptions that may be challenged in the context of impaired kidney physiology. To further evaluate the potential influence of urine dilution adjustment, we performed a complementary sensitivity analysis using non-creatinine-adjusted urinary element concentrations with urinary creatinine included as a covariate. The findings were materially unchanged, suggesting that the observed associations were not solely attributable to creatinine ratio standardization. Nevertheless, because urinary creatinine is itself influenced by kidney function and other physiological factors, neither ratio standardization nor covariate adjustment completely eliminates the possibility of residual bias. We therefore retained creatinine-adjusted concentrations to maximize comparability with the existing urinary biomonitoring literature, while emphasizing that urinary element concentrations in individuals with CKD should be interpreted as markers of both exposure and kidney excretory capacity, rather than as direct indicators of causal effects. To facilitate interpretation of these findings in the context of a cross-sectional design, a simplified conceptual framework is provided in Supplementary Figure S5.

### 4.1 Possible mechanisms

Although impaired kidney function is generally characterized by reduced glomerular filtration and altered tubular handling, urinary concentrations of different elements do not necessarily change in a uniform direction. The observed heterogeneous patterns likely reflect element-specific kidney handling, including differences in filtration, tubular reabsorption and secretion, protein binding, biological half-lives, and homeostatic regulation. Consequently, reduced urinary concentrations may reflect diminished excretory capacity or kidney retention for some elements, whereas increased urinary concentrations, particularly for essential trace elements, may indicate impaired tubular reabsorption or disrupted homeostatic control rather than increased exposure.

#### 4.1.1 Nephrotoxic metals: reverse causality and concentration-based bias

Several nephrotoxic metals, including Cd, Hg, Pb, and Tl, were inversely associated with CKD status in our study. These findings, although initially counterintuitive, may reflect altered kidney handling or decreased urinary elimination capacity in individuals with reduced kidney function. As glomerular and tubular damage progresses and kidney filtration and secretion capacity decline, urinary concentrations may decrease despite possible systemic accumulation. This pattern likely reflects reverse causality rather than protective effects.

In addition, all urinary concentrations were standardized to urinary creatinine to account for urine dilution. However, because urinary creatinine excretion is altered in CKD, creatinine ratio standardization may introduce differential bias across levels of kidney function. Depending on the relative changes in urinary creatinine and element excretion, creatinine standardization may either attenuate or exaggerate the observed associations. Accordingly, creatinine-standardized urinary element concentrations should be interpreted cautiously in populations with impaired kidney function.

From a biological perspective, heavy metals such as Cd and Pb are known to accumulate in the kidney cortex and exert nephrotoxic effects through multiple pathways, including oxidative stress, lipid peroxidation, mitochondrial dysfunction, impaired DNA repair, decreased antioxidant capacity, apoptosis, proximal tubular dysfunction, interstitial fibrosis, tubular atrophy, and reductions in eGFR (Xu et al., 2018). As kidney damage worsens, the resulting decrease in filtration and secretion may further suppress urinary element elimination, reinforcing the apparent inverse associations.

Our interpretation is consistent with findings from previous cross-sectional studies. For instance, studies conducted in Asia and the Americas reported significant positive associations between urinary Cd and Pb concentrations and eGFR (Buser et al., 2016; Wang et al., 2016). Additionally, a study from Mexico indicated weak positive correlations between urinary Cd and Tl levels and eGFR (Weaver et al., 2014). Research on adolescents further demonstrated that mixed exposure to Hg, Cd, and Pb was significantly positively associated with eGFR (Sanders et al., 2019).

#### 4.1.2 Essential trace elements: altered homeostasis and nonlinear responses

Se, an essential antioxidant trace element, was inversely associated with CKD status. The reduced urinary Se observed in CKD patients may reflect impaired kidney filtration or altered tubular handling, rather than decreased systemic availability or intake. Previous studies have similarly noted a decrease in urinary Se with worsening kidney function (Shen et al., 2023), suggesting an inverse association between urinary Se and CKD status.

Mo, another essential trace element, was also inversely associated with CKD. As Mo is filtered by the glomerulus and reabsorbed in the proximal tubules, reduced urinary concentrations in CKD may reflect both lower filtration and impaired reabsorption. Consistent with this, the study by Jin et al. (2018) reported that decreased kidney function was associated with decreased urinary Mo. Importantly, reduced urinary Mo does not necessarily imply reduced systemic Mo exposure. Emerging evidence suggests that disturbances in Mo homeostasis may have implications beyond kidney function. For example, a recent study in BRCA1 mutation carriers reported that higher blood Mo concentrations were associated with increased risks of ovarian cancer and overall cancer (Matuszczak et al., 2024). Together, these findings suggest that lower urinary Mo in CKD may reflect disease-related retention or altered systemic distribution rather than lower exposure, underscoring the need to carefully distinguish between exposure, excretion, and disease-related retention when interpreting urinary Mo biomarkers.

In contrast, Zn showed a strong and consistent positive association with CKD, both in single-element models and within urinary element mixture analyses. This finding is consistent with previous studies reporting elevated urinary Zn concentrations and reduced plasma Zn levels in CKD patients (Damianaki et al., 2020; Yu et al., 2023). These patterns suggest impaired tubular reabsorption, possibly due to structural loss of proximal tubule mass, altered expression of Zn transporters, or proteinuria-mediated Zn wasting (Bovio et al., 2007; Kambe et al., 2015). Accordingly, higher urinary zinc concentrations should not be interpreted as indicating higher zinc exposure or toxicity. Instead, disruption of Zn homeostasis in CKD is more likely to reflect impaired kidney handling, particularly tubular dysfunction or reduced reabsorption capacity. From a clinical and public health perspective, elevated urinary Zn may therefore serve as a nonspecific indicator of tubular injury or altered kidney function, rather than a marker of Zn intake or toxicity.

### 4.2 Strengths and limitations

This study has several notable strengths. It utilized a large, population-based cohort and multiple analytical approaches, including logistic regression, RCS, and mixture models (WQS, qgcomp, and BKMR), which yielded generally consistent findings across complementary analytical approaches. Furthermore, this cross-sectional analysis complements previous prospective findings from the same cohort by offering insight into how urinary element concentrations may vary in individuals with established CKD.

However, the cross-sectional design limits causal inference. The observed associations may reflect potential reverse causality, particularly for nephrotoxic elements whose urinary concentrations decline in the setting of impaired kidney function. Moreover, CKD was operationally defined based on a single eGFR measurement obtained at the study visit. Because repeated eGFR measurements at least 3 months apart were unavailable, persistent kidney dysfunction according to KDIGO criteria could not be confirmed, and some degree of outcome misclassification cannot be excluded. Residual confounding cannot be excluded because detailed information on dietary intake, nutritional supplement use, occupational exposure, seafood consumption, smoking intensity, and most medication use was unavailable. Although participants receiving lithium or iodine treatment were excluded because these medications may directly influence urinary element concentrations, information on other medications was unavailable. Moreover, hypertension and diabetes were included as established risk factors for CKD, although they may also partially lie on the causal pathway underlying the association between urinary element concentrations and CKD.

In addition, LASSO-based variable selection was performed using the same outcome data that were subsequently analysed. Therefore, the conventional confidence intervals and P values reported in downstream analyses may not fully account for the variable-selection process and should be interpreted as exploratory rather than confirmatory. Given the large number of single-element, non-linear, and mixture analyses performed, the possibility of false-positive findings due to multiple comparisons cannot be excluded. Because the primary objective of this study was exploratory, these findings should be interpreted as hypothesis-generating and require confirmation in independent studies.

Although the primary multivariable models had approximately 12 events per degree of freedom, the relatively limited number of participants with prevalent CKD in relation to the complexity of the mixture models may have reduced statistical precision. Therefore, despite generally consistent findings across WQS regression, qgcomp, and BKMR, these mixture analyses should be regarded as exploratory and interpreted cautiously.

Like most biomonitoring studies, our reliance on spot urine samples may be subject to measurement error and may not fully reflect long-term internal dose or total body burden for all elements, especially those with complex pharmacokinetics or high tissue affinity. Finally, urinary concentrations were standardized to creatinine to account for urine dilution; however, urinary creatinine excretion differs by kidney function, which may introduce differential standardization across CKD status.

## 5 Conclusion

In this cross-sectional analysis, participants with CKD had lower urinary concentrations of several known nephrotoxic elements, including cadmium, mercury, lead, and thallium, compared with those without CKD, consistent with altered kidney handling and reduced urinary excretion. In contrast, urinary zinc concentrations were higher among individuals with CKD, suggesting disruptions in trace element homeostasis. Overall, this study provides an interpretive framework for understanding urinary element biomarkers in CKD, highlighting that urinary concentrations may reflect both exposure and excretory function and that potential reverse causality should be carefully considered when using urinary elements to study kidney outcomes. Additional longitudinal analyses and tissue-based biomarker studies may further clarify temporal relationships and strengthen the interpretation of urinary element biomarkers in kidney disease.

## Data Availability

The datasets presented in this article are not readily available because the data from the CoLaus|PsyCoLaus study cannot be fully shared as they contain potentially sensitive personal information on participants. According to the Ethics Committee for Research of the Canton of Vaud, sharing these data would violate Swiss privacy protection laws. However, coded individual-level data that do not allow identification of participants are available upon request to researchers who meet the data sharing criteria of the CoLaus|PsyCoLaus Datacenter (CHUV, Lausanne, Switzerland). Requests to access the datasets should be directed to Researchers who wish to request access to the data used in this study should contact the CoLaus|PsyCoLaus data center via research.colaus@chuv.ch or research.psycolaus@chuv.ch.
