# Supplementary material for: Urinary heavy metal and trace element mixtures in chronic kidney disease: associations and implications for potential reverse causality
Source: Front Toxicol. 2026 Jul 29;8:1892052. doi: 10.3389/ftox.2026.1892052 (PMC13461065; doi:10.3389/ftox.2026.1892052)
Supplement: Supplementary file 1 [file Supplementaryfile1.docx]

**Supplementary information**

**Supplementary Materials: Methods**

## ***2.4. Covariates***

Education was categorized into high (university), middle (high school) and low (apprenticeship + mandatory). Marital status was defined as living alone (single, divorced, widowed) or living with a partner. Usual alcohol consumption during the week was self-reported and reported as the number of units (glasses of wine, bottles or cans of beer, and shots of spirits) per week. Smoking was self-reported and categorized as never, former (irrespective of the time since quitting smoking) and current.

Blood pressure (BP) was measured using an Omron® HEM-907 automated oscillometric sphygmomanometer after at least a 10-minute rest in a seated position, and the average of the last two measurements was used. Hypertension was defined by a SBP ≥140 mm Hg or a DBP ≥90 mm Hg or presence of antihypertensive drug treatment. Glucose was assessed by glucose dehydrogenase. Diabetes mellitu was defined as fasting plasma glucose ≥7.0 mmol/L and/or presence of oral hypoglycaemic or insulin treatment. Body weight and height were measured with participants barefoot and in light indoor clothes. Body weight was measured in kilograms to the nearest 100 g using a Seca® scale (Hamburg, Germany). Height was measured to the nearest 5 mm using a Seca® (Hamburg, Germany) height gauge. Body mass index was calculated and categorized as normal (<25 kg/m^2^), overweight ≥25 and <30 kg/m^2^) and obese ≥30 kg/m^2^).

Participants reported all medicines prescribed or bought over the counter. Medicines were coded according to the Anatomic, Therapeutic and Chemical classification of the WHO. Lithium treatment was defined by codes N05AN* and D11AX04. Iodine containing drugs were defined by codes H03C*, D08AG* and H03AA*, where *=any code.

***2.7. Statistical analysis***

We applied Bayesian Kernel Machine Regression (BKMR) to evaluate the joint effects of urinary elements on CKD status, allowing for potential non-linear and interactive associations among mixture components. BKMR analyses were conducted using the bkmr package in R with a logistic link for the binary CKD outcome (family = "binomial"). All exposure variables were standardized using Z-score transformation (scale()) before modeling. Covariates included sex, age, education level, marital status, alcohol consumption, smoking status, hypertension, diabetes, and body mass index. Variable selection was enabled (varsel = TRUE), and posterior inclusion probabilities (PIPs) were extracted using ExtractPIPs(). Markov chain Monte Carlo (MCMC) sampling was run for 10,000 iterations (iter = 10,000) with estimation of the kernel parameter enabled (est.h = TRUE). No explicit burn-in period or additional thinning was applied. Default prior distributions implemented in the bkmr package were used. To improve computational efficiency, 50 knots were specified (nd = 50) using a space-filling design approach (fields::cover.design), and the resulting knot locations were passed to kmbayes via the knots argument. The default Gaussian kernel implemented in the bkmr package was used. Model convergence was assessed by visual inspection of trace plots for key parameters, including regression coefficients (TracePlot, par = "beta"), residual variance (par = "sigsq.eps"), and the kernel parameter (par = "r"). Effective sample sizes (ESS) for representative model parameters were calculated using the coda package and are reported in **Supplementary Table 11**. Representative trace plots are shown in **Supplementary Figure 6**.

**Supplementary Table 1:** Distribution of participants by KDIGO glomerular filtration rate (GFR) categories at baseline. CoLaus|PsyCoLaus study, Lausanne, Switzerland.

| **KDIGO G-stage** | **eGFR(mL/min/1.73m²)** | **n (%)** |
| --- | --- | --- |
| G1 | ≥90 | 3215 (51.92) |
| G2 | 60–89 | 2806 (45.32) |
| G3a | 45–59 | 148 (2.39) |
| G3b | 30–44 | 15 (0.24) |
| G4 | 15–29 | 5 (0.08) |
| G5 | <15 | 3 (0.05) |

CKD in this study was defined as eGFR < 60 mL/min/1.73 m². KDIGO GFR categories are presented for descriptive purposes only. KDIGO: Kidney Disease: Improving Global Outcomes.

**Supplementary Table 2:** Comparison between excluded and included participants. CoLaus|PsyCoLaus study, Lausanne, Switzerland.

| **Variables** | **Included (n=6192)** | **Excluded (n=541)** | ***P* value** |
| --- | --- | --- | --- |
| Age, years | 52.4 ± 10.7 | 55.4 ± 10.8 | <0.001 |
| Female, % | 3202 (51.7) | 342 (63.2) | <0.001 |
| Education level, % |  |  | 0.337 |
| High | 1225 (19.8) | 95 (18.0) |  |
| Middle | 1505 (24.3) | 120 (22.8) |  |
| Low | 3462 (55.9) | 312 (59.2) |  |
| Marital status, % |  |  | 0.326 |
| Living alone | 2028 (32.8) | 186 (34.8) |  |
| Living in couple | 4164 (67.2) | 348 (65.2) |  |
| Smoking status, % |  |  | 0.120 |
| Never | 2494 (40.3) | 238 (44.5) |  |
| Former | 2014 (32.5) | 169 (31.6) |  |
| Current | 1684 (27.2) | 128 (23.9) |  |
| Alcohol consumption (%) |  |  | 0.005 |
| None | 1734 (28.0) | 181 (33.5) |  |
| 1-13/week | 3362 (54.3) | 291 (53.8) |  |
| 14-27/week | 856 (13.8) | 57 (10.5) |  |
| 28+/week | 240 (3.9) | 12 (2.2) |  |
| BMI group, % |  |  | 0.522 |
| Normal | 2989 (48.3) | 248 (46.1) |  |
| Overweight | 2262 (36.5) | 200 (37.2) |  |
| Obese | 941 (15.2) | 90 (16.7) |  |
| Hypertension, % | 2257 (36.5) | 243 (45.5) | <0.001 |
| Diabetes, % | 402 (6.5) | 34 (6.5) | 0.989 |
| Serum creatinine, µmol/L | 79.8 ± 19.7 | 79.8 ± 34.2 | 0.940 |
| Uric acid, µmol/L | 312.4 ± 84.8 | 311.8 ± 84.3 | 0.883 |
| Kidney function (%) |  |  | 0.001 |
| Non-CKD | 6021 (97.2) | 496 (94.7) |  |
| CKD | 171 (2.8) | 28 (5.3) |  |
| eGFR, mL/min/1.73 m^2^ | 89.8 ± 15.1 | 86.6 ± 16.3 | <0.001 |

Results are expressed as number of participants (column percentage) for categorical variables and as average ± standard deviation for continuous variables. Between-group comparisons were performed using chi-square for categorical variables and Student’s t-test for continuous variables. Abbreviations: BMI: body mass index; CKD: chronic kidney disease; eGFR: estimated glomerular filtration rate.

**Supplementary Table 3:** Concentration, number, and percentage of LOD for all 23 elements. CoLaus|PsyCoLaus study, Lausanne, Switzerland.

| **Elements** | **LOD (ug/** **g creatinine)** | **Number of observations below LOD (n and %)** | |
| --- | --- | --- | --- |
| Li | 0.437 | 0 | 0 |
| Be | 0.002 | 4814 | 77.75 |
| Al | 1.223 | 525 | 8.48 |
| V | 0.024 | 6 | 0.10 |
| Cr | 0.100 | 1066 | 17.22 |
| Mn | 0.568 | 5449 | 88.00 |
| Co | 0.023 | 2 | 0.03 |
| Ni | 0.044 | 7 | 0.11 |
| Cu | 2.712 | 10 | 0.16 |
| Zn | 8.596 | 0 | 0 |
| As | 0.117 | 0 | 0 |
| Se | 1.578 | 0 | 0 |
| Mo | 0.054 | 0 | 0 |
| Ag | 0.050 | 5089 | 82.19 |
| Cd | 0.042 | 0 | 0 |
| Sn | 0.152 | 210 | 3.39 |
| Sb | 0.044 | 3094 | 49.97 |
| Hg | 0.094 | 21 | 0.34 |
| Pb | 0.226 | 28 | 0.45 |
| Tl | 0.003 | 0 | 0 |
| Bi | 0.100 | 6037 | 97.50 |
| Fe | 0.080 | 3 | 0.05 |
| I | 2.497 | 0 | 0 |

**Supplementary Table 4:** Abbreviations, full names and distribution of the 23 elements. CoLaus|PsyCoLaus study, Lausanne, Switzerland.

| **Elements** | **Full names** | **Median** | **Interquartile Range (IQR)** |
| --- | --- | --- | --- |
| Li (ug/g creatinine) | Lithium | 17.20 | 11.44-29.18 |
| Be (ng/g creatinine) | Beryllium | 1.03 | 0.66-1.86 |
| Al (ug/g creatinine) | Aluminum | 4.17 | 2.39-7.34 |
| V (ng/g creatinine) | Vanadium | 124.09 | 88.35-183.44 |
| Cr (ug/g creatinine) | Chromium | 0.20 | 0.12-0.33 |
| Mn (ug/g creatinine) | Manganese | 0.22 | 0.16-0.34 |
| Co (ug/g creatinine) | Cobalt | 0.16 | 0.11-0.27 |
| Ni (ug/g creatinine) | Nickel | 0.97 | 0.65-1.51 |
| Cu (ug/g creatinine) | Copper | 8.98 | 7.35-11.26 |
| Zn (ug/g creatinine) | Zinc | 297.70 | 197.55-431.44 |
| As (ug/g creatinine) | Arsenic | 15.28 | 7.52-36.70 |
| Se (ug/g creatinine) | Selenium | 42.59 | 35.21-51.31 |
| Mo (ug/g creatinine) | Molybdenum | 29.43 | 19.69-41.42 |
| Ag (ng/g creatinine) | Silver | 25.87 | 18.31-40.15 |
| Cd (ug/g creatinine) | Cadmium | 0.43 | 0.26-0.70 |
| Sn (ug/g creatinine) | Tin | 0.54 | 0.33-0.91 |
| Sb (ng/g creatinine) | Antimony | 44.00 | 28.36-69.72 |
| Hg (ug/g creatinine) | Mercury | 0.97 | 0.56-1.68 |
| Pb (ug/g creatinine) | Lead | 1.30 | 0.87-1.97 |
| TI (ug/g creatinine) | Thallium | 0.18 | 0.13-0.24 |
| Bi (ng/g creatinine) | Bismuth | 4.55 | 3.26-7.21 |
| Fe (ug/g creatinine) | Iron | 4.58 | 3.14-7.27 |
| I (ug/g creatinine) | Iodine | 91.94 | 71.02-121.89 |

**Supplementary Table 5.** The final selected 18 elements included in the main analyses and their distributions. CoLaus|PsyCoLaus study, Lausanne, Switzerland.

| **Elements** | **Median** | **Interquartile Range** | **LOD** | **Below LOD (n and %)** |
| --- | --- | --- | --- | --- |
| Li (ug/g creatinine) | 17.20 | 11.44-29.18 | 0.437 | 0 (0) |
| Al (ug/g creatinine) | 4.17 | 2.39-7.34 | 1.223 | 525 (8.48) |
| V (ng/g creatinine) | 124.09 | 88.35-183.44 | 0.024 | 6 (0.10) |
| Cr (ug/g creatinine) | 0.20 | 0.12-0.33 | 0.100 | 1066 (17.22) |
| Co (ug/g creatinine) | 0.16 | 0.11-0.27 | 0.023 | 2 (0.03) |
| Cu (ug/g creatinine) | 8.98 | 7.35-11.26 | 2.712 | 10 (0.16) |
| Zn (ug/g creatinine) | 297.70 | 197.55-431.44 | 8.596 | 0 (0) |
| As (ug/g creatinine) | 15.28 | 7.52-36.70 | 0.117 | 0 (0) |
| Se (ug/g creatinine) | 42.59 | 35.21-51.31 | 1.578 | 0 (0) |
| Mo (ug/g creatinine) | 29.43 | 19.69-41.42 | 0.054 | 0 (0) |
| Cd (ug/g creatinine) | 0.43 | 0.26-0.70 | 0.042 | 0 (0) |
| Sn (ug/g creatinine) | 0.54 | 0.33-0.91 | 0.152 | 210 (3.39) |
| Sb (ng/g creatinine) | 44.00 | 28.36-69.72 | 0.044 | 3094 (49.97) |
| Hg (ug/g creatinine) | 0.97 | 0.56-1.68 | 0.094 | 21 (0.34) |
| Pb (ug/g creatinine) | 1.30 | 0.87-1.97 | 0.226 | 28 (0.45) |
| TI (ug/g creatinine) | 0.18 | 0.13-0.24 | 0.003 | 0 (0) |
| Fe (ug/g creatinine) | 4.58 | 3.14-7.27 | 0.080 | 3 (0.05) |
| I (ug/g creatinine) | 91.94 | 71.02-121.89 | 2.497 | 0 (0) |

**Supplementary Table 6:** Sensitivity analysis of associations between non-creatinine-adjusted urinary elements and CKD status. CoLaus|PsyCoLaus study, Lausanne, Switzerland.

| **Elements** | **OR (95% CI)** | ***P* value** |
| --- | --- | --- |
| Li | 1.00 (0.84, 1.20) | 0.991 |
| Al | 1.03 (0.87, 1.21) | 0.754 |
| V | 0.85 (0.66, 1.11) | **0.001** |
| Cr | 1.10 (0.91, 1.31) | 0.323 |
| Co | 1.06 (0.90, 1.26) | 0.472 |
| Cu | 1.05 (0.81, 1.36) | 0.709 |
| Zn | 1.22 (0.99, 1.50) | 0.059 |
| As | 1.01 (0.89, 1.15) | 0.822 |
| Se | 0.78 (0.60, 1.02) | 0.073 |
| Mo | 0.81 (0.65, 1.01) | 0.058 |
| Cd | 0.78 (0.63, 0.95) | **0.015** |
| Sn | 1.10 (0.93, 1.30) | 0.259 |
| Sb | 0.94 (0.77, 1.15) | 0.544 |
| Hg | 0.78 (0.66, 0.93) | **0.004** |
| Pb | 0.69 (0.56, 0.85) | **<0.001** |
| Tl | 0.62 (0.48, 0.81) | **<0.001** |
| Fe | 1.04 (0.87, 1.25) | 0.645 |
| I | 1.19 (0.95, 1.48) | 0.126 |

Multivariable logistic regression models were used for the analysis. Results are presented as odds ratios (ORs) with corresponding 95% confidence intervals. The models were adjusted for the following variables: age (continuous), sex (male or female), BMI (normal, overweight, or obese), education (low, medium, high), marital status (alone or in a couple), smoking status (never, former, current), alcohol consumption (categorized into four groups), hypertension (yes or no), and diabetes (yes or no).

**Supplementary Table 7:** Sensitivity analysis of associations between urinary elements and CKD status using non-creatinine-adjusted urinary element concentrations with urinary creatinine included as a covariate. CoLaus|PsyCoLaus study, Lausanne, Switzerland.

| **Elements** | **OR (95% CI)** | ***P* value** |
| --- | --- | --- |
| Li | 0.98 (0.81, 1.19) | 0.821 |
| Al | 1.01 (0.85, 1.20) | 0.930 |
| V | 0.77 (0.57, 1.04) | 0.093 |
| Cr | 1.08 (0.90, 1.31) | 0.415 |
| Co | 1.04 (0.86, 1.26) | 0.673 |
| Cu | 0.97 (0.67, 1.40) | 0.857 |
| Zn | 1.28 (0.99, 1.67) | 0.061 |
| As | 1.00 (0.87, 1.15) | 0.965 |
| Se | 0.28 (0.16, 0.48) | **<0.001** |
| Mo | 0.66 (0.50, 0.87) | **0.003** |
| Cd | 0.56 (0.42, 0.75) | **<0.001** |
| Sn | 1.09 (0.90, 1.32) | 0.373 |
| Sb | 0.88 (0.70, 1.11) | 0.291 |
| Hg | 0.70 (0.58, 0.85) | **<0.001** |
| Pb | 0.49 (0.37, 0.65) | **<0.001** |
| Tl | 0.41 (0.29, 0.58) | **<0.001** |
| Fe | 1.02 (0.83, 1.24) | 0.877 |
| I | 1.20 (0.93, 1.54) | 0.170 |

Multivariable logistic regression models were used to evaluate the associations between urinary elements and CKD status using non-creatinine-adjusted urinary element concentrations, with urinary creatinine included as a covariate to account for urine dilution. Results are presented as odds ratios (ORs) with corresponding 95% confidence intervals (CIs). Models were adjusted for age (continuous), sex (male or female), BMI (normal, overweight, or obese), education (low, medium, high), marital status (alone or in a couple), smoking status (never, former, current), alcohol consumption (categorized into four groups), hypertension (yes or no), diabetes (yes or no), and urinary creatinine (continuous).

**Supplementary Table 8:** Sensitivity analysis of associations between urinary elements and CKD status using a strict healthy control group. CoLaus|PsyCoLaus study, Lausanne, Switzerland.

| **Elements** | **OR (95% CI)** | ***P* value** |
| --- | --- | --- |
| Li | 0.90 (0.71, 1.13) | 0.363 |
| Al | 0.91 (0.73, 1.13) | 0.408 |
| V | 0.57 (0.40, 0.81) | **0.002** |
| Cr | 1.02 (0.82, 1.28) | 0.851 |
| Co | 0.97 (0.75, 1.27) | 0.838 |
| Cu | 1.03 (0.67, 1.60) | 0.888 |
| Zn | 1.69 (1.23, 2.32) | 0.001 |
| As | 0.93 (0.78, 1.12) | 0.452 |
| Se | 0.18 (0.09, 0.36) | **<0.001** |
| Mo | 0.56 (0.40, 0.78) | **0.001** |
| Cd | 0.39 (0.26, 0.58) | **<0.001** |
| Sn | 1.03 (0.82, 1.29) | 0.805 |
| Sb | 0.86 (0.64, 1.15) | 0.304 |
| Hg | 0.56 (0.44, 0.73) | **<0.001** |
| Pb | 0.39 (0.27, 0.55) | **<0.001** |
| Tl | 0.27 (0.18, 0.41) | **<0.001** |
| Fe | 1.23 (0.96, 1.57) | 0.104 |
| I | 1.18 (0.85, 1.64) | 0.332 |

Multivariable logistic regression models were used to evaluate the associations between urinary elements and CKD status in a sensitivity analysis restricting the control group to strictly healthy participants. CKD cases were defined as in the main analysis. Healthy controls were defined as participants without CKD, with eGFR ≥ 90 mL/min/1.73 m², and without hypertension or diabetes. Results are presented as odds ratios (ORs) with corresponding 95% confidence intervals and P values for trends. The models were adjusted for the following variables: age (continuous), sex (male or female), BMI (normal, overweight, or obese), education (low, medium, high), marital status (alone or in a couple), smoking status (never, former, current), and alcohol consumption (categorized into four groups).

**Supplementary Table 9:** Multivariable linear regression analyses of associations between CKD status and urinary element concentrations. CoLaus|PsyCoLaus study, Lausanne, Switzerland.

| **Elements** | **β (95% CI)** | ***P* value** |
| --- | --- | --- |
| Li | -0.04 (-0.17, 0.10) | 0.609 |
| Al | 0.00 (-0.15, 0.14) | 0.974 |
| V | -0.09 (-0.18, 0.01) | 0.080 |
| Cr | 0.05 (-0.08, 0.18) | 0.421 |
| Co | 0.04 (-0.08, 0.15) | 0.531 |
| Cu | -0.01 (-0.07, 0.06) | 0.832 |
| Zn | 0.09 (0.00, 0.19) | **0.048** |
| As | -0.02 (-0.20, 0.16) | 0.824 |
| Se | -0.11 (-0.16, -0.06) | **<0.001** |
| Mo | -0.12 (-0.21, -0.03) | **0.010** |
| Cd | -0.21 (-0.30, -0.12) | **<0.001** |
| Sn | 0.05 (-0.07, 0.18) | 0.398 |
| Sb | -0.08 (-0.19, 0.04) | 0.187 |
| Hg | -0.28 (-0.41, -0.16) | **<0.001** |
| Pb | -0.27 (-0.37, -0.18) | **<0.001** |
| Tl | -0.20 (-0.27, -0.12) | **<0.001** |
| Fe | 0.00 (-0.12, 0.13) | 0.947 |
| I | 0.06 (-0.02, 0.13) | 0.173 |

To complement the primary analyses, multivariable linear regression models were performed with log-transformed, creatinine-adjusted urinary element concentrations as the dependent variables and CKD status as the independent variable. Results are presented as regression coefficients (β) with corresponding 95% confidence intervals (CIs) and P values. The models were adjusted for the following variables: age (continuous), sex (male or female), BMI (normal, overweight, or obese), education (low, medium, high), marital status (alone or in a couple), smoking status (never, former, current), alcohol consumption (categorized into four groups), hypertension (yes or no), and diabetes (yes or no).

**Supplementary Table 10:** Inverse probability weighted sensitivity analysis of associations between urinary element concentrations and CKD status. CoLaus|PsyCoLaus study, Lausanne, Switzerland.

| **Elements** | **OR (95% CI)** | ***P* value** |
| --- | --- | --- |
| Li | 0.96 (0.79, 1.16) | 0.660 |
| Al | 0.99 (0.83, 1.17) | 0.906 |
| V | 0.77 (0.57, 1.04) | 0.088 |
| Cr | 1.05 (0.85, 1.28) | 0.653 |
| Co | 1.04 (0.84, 1.28) | 0.726 |
| Cu | 0.94 (0.59, 1.50) | 0.784 |
| Zn | 1.27 (1.01, 1.61) | **0.045** |
| As | 1.00 (0.87, 1.15) | 0.957 |
| Se | 0.27 (0.16, 0.46) | **<0.001** |
| Mo | 0.65 (0.50, 0.86) | **0.002** |
| Cd | 0.56 (0.41, 0.77) | **<0.001** |
| Sn | 1.08 (0.89, 1.32) | 0.445 |
| Sb | 0.87 (0.70, 1.08) | 0.210 |
| Hg | 0.70 (0.58, 0.85) | **<0.001** |
| Pb | 0.49 (0.36, 0.67) | **<0.001** |
| Tl | 0.44 (0.29, 0.66) | **<0.001** |
| Fe | 0.99 (0.80, 1.24) | 0.955 |
| I | 1.15 (0.84, 1.58) | 0.392 |

Inverse probability weighted logistic regression models were used to evaluate the associations between urinary element concentrations and CKD status. Stabilized inverse probability weights were estimated based on the probability of inclusion in the final analytic sample using observed baseline characteristics. Results are presented as odds ratios (ORs) with corresponding 95% confidence intervals (CIs) and P values. Models were adjusted for age (continuous), sex (male or female), BMI (normal, overweight, or obese), education (low, medium, high), marital status (alone or in a couple), smoking status (never, former, current), alcohol consumption (categorized into four groups), hypertension (yes or no), and diabetes (yes or no).

**Supplementary Table 11:** Effective sample sizes (ESS) for representative BKMR model parameters.

| **Parameter** | **Minimum ESS** | **Median ESS** |
| --- | --- | --- |
| β | 10.9 | 55.6 |
| λ | 57.3 | 57.3 |
| r | 2.79 | 9.98 |

ESS, effective sample size. ESS values were calculated from posterior MCMC samples using the coda package in R. Minimum and median ESS are presented for representative model parameters (β, λ, and r).

**Supplementary Figure 1:** Selection of participants from Colaus 2003–2006. CoLaus|PsyCoLaus study, Lausanne, Switzerland.

**Supplementary Figure 2:** Pearson correlation between urinary elements. CoLaus|PsyCoLaus study, Lausanne, Switzerland.

Pearson correlation matrix among 18 urinary elements, based on log-transformed, creatinine-adjusted concentrations. Only the lower triangle is displayed. Circle size and color intensity represent the strength of correlation.

**Supplementary Figure 3:** Univariate exposure–response analysis between urinary element concentrations and CKD status. CoLaus|PsyCoLaus study, Lausanne, Switzerland.

Univariate exposure–response relationships between individual urinary element concentrations and CKD status, estimated using the BKMR model. Each panel shows the estimated exposure–response relationship of a single urinary element with CKD status, expressed as the change in CKD log-odds, while holding all other urinary element concentrations constant at their median levels. Solid blue lines represent posterior means, and shaded areas indicate 95% credible intervals.

**Supplementary Figure 4:** Bivariate dose-response analysis between urinary element mixtures and CKD status. CoLaus|PsyCoLaus study, Lausanne, Switzerland.

Bivariate concentration–response relationships between urinary elements and CKD status, estimated using the BKMR model. Each cell shows the estimated change in CKD log-odds associated with one element while fixing a second element at the 25th (red), 50th (green), or 75th (blue) percentile. Diagonal panels represent univariate associations for reference.

**Supplementary Figure 5:** Conceptual framework for interpreting urinary element concentrations in relation to CKD status.

Conceptual framework illustrating the relationships among kidney function (eGFR), urinary element concentrations, and CKD status in this cross-sectional study. The diagram emphasizes that urinary element concentrations are biomarkers influenced by kidney excretory function and urine dilution/creatinine excretion, and that differences observed by CKD status may therefore reflect altered kidney handling and reverse causality rather than protective effects. Covariates represent demographic, lifestyle, and clinical factors included for adjustment. The covariates were selected a priori based on previous epidemiological evidence and biological plausibility. Hypertension and diabetes were included because they are established determinants of kidney function. However, they may also partially lie on the causal pathway underlying the association between urinary element concentrations and CKD. This diagram is intended as an interpretive framework and does not represent a formal directed acyclic graph for causal inference.

**Supplementary Figure 6:** Trace plot of a representative regression coefficient (β) from the BKMR model.

The trace plot was used to assess MCMC convergence and mixing and showed stable sampling behavior without obvious trends.
